# Calculation of Austenite Generalized Stacking Fault Energy in M50NiL Steel

**DOI:** 10.3390/ma19061170

**Published:** 2026-03-17

**Authors:** Zifeng Ding, Jiaxu Guo, Lina Zhou, Xinghong Zhang, Xinxin Ma

**Affiliations:** 1 School of Materials Science & Engineering, Harbin Institute of Technology, Harbin 150001, China; 21b909019@stu.hit.edu.cn (Z.D.); jxguo_hit@163.com (J.G.); 2 AECC Harbin Bearing Co., Ltd., Harbin 150025, China; zhoulina626@126.com (L.Z.); zhxh710@126.com (X.Z.)

**Keywords:** M50NiL steel, first-principles calculations, austenite, generalized stacking fault energy, twinning

## Abstract

By optimizing the carburizing heat treatment process, the grain size of the carburized layer of M50NiL steel was successfully refined to the sub-micron level. The mechanism for the generation of a large number of sub-micron crystal regions (SMCR) is that dislocations are entangled and linked due to the pinning effect of nanometer-sized carbides. In this study, a stacking fault energy (SFE) model for austenite in M50NiL steel was established. First-principles calculations were employed to investigate the effects of alloying elements, as well as the position and quantity of carbon (C) atoms, on the generalized stacking fault energy (GSFE). The variations in SFE were further analyzed in combination with differential charge density calculations. The simulation results revealed that the addition of alloying elements excluding nickel led to a reduction in the unstable stacking fault energy. Differential charge density analysis indicated that this decrease was associated with the weakening of Fe–Fe bonds in the L0 layer, where stacking faults occurred. When C atoms are interstitially dissolved near the L0 layer, the Fe–Fe bonds near the L0 layer are enhanced, and the unstable stacking fault energy is correspondingly increased. Compared with the pure iron system, the combined effect of alloying elements and C atoms in M50NiL steel maintained a relatively low level of both the unstable stacking fault energy and the stacking fault formation barrier, provided that C atoms were not dissolved in the L1 layer. This condition was favorable for dislocation slip. Meanwhile, the stable stacking fault energy significantly increased, enhancing the stability of austenite. Based on these simulation results, the relationship between the GSFE of austenite in M50NiL steel and the formation of subgrains and twins within the submicron crystalline regions of the carburized layer was discussed.

## 1. Introduction

M50NiL steel exhibits excellent surface hardness and toughness after carburizing, making it the material of choice for aeroengine bearings. Its heat treatment process typically includes carburizing, quenching, cryogenic treatment, and tempering [1,2,3,4,5,6,7]. In our previous study, by extending the carburizing duration, large grain-boundary M7C3 carbides were formed, which act as particle-stimulated nucleation (PSN) sites during quenching, thereby facilitating the recrystallization of retained austenite (RA). Simultaneously, a high density of subgrains was formed within the carburized layer, effectively refining the RA grains near the surface to the sub-micron scale [8]. Subsequent cryogenic and tempering treatments, influenced by the refined RA grain size, further contributed to the refinement of the martensite grains. Moreover, numerous twins were generated within the sub-micron crystalline regions. This grain refinement strategy ultimately reduces the wear of the carburized layer by nearly 50%.

The driving force for recrystallization and subgrain formation in this method stems from the release of residual stresses accumulated during carburizing, which are subsequently relieved during quenching. The mechanism of subgrain formation involves dislocation entanglement during their movement. These dislocations form subgrain boundaries due to the pinning effect exerted by nanoscale carbides within the original austenite grains. In the carburized specimens, the surface layer retains a residual stress of approximately 500 MPa at room temperature after quenching at 1100 °C. Under these conditions, the combination of high stress and elevated temperature significantly enhances atomic mobility and intensifies lattice vibrations. This facilitates dislocation movement by making it easier for dislocations to break free from the constraints of surrounding atomic bonds [9,10]. Additionally, high temperature reduces the energy barrier for dislocation movement [11]. Beyond these factors, numerous studies have indicated that stacking fault energy (SFE) plays a crucial role in dislocation behavior. It is also closely associated with the formation of deformation twins [12,13,14,15,16,17,18,19,20].

Stacking fault energy (SFE) refers to the energy per unit area associated with the displacement of atomic layers in close-packed crystal structures. In face-centered cubic (FCC) structures, atomic planes are normally arranged in an ABCABC… sequence, whereas a deviation from this order, such as an ABAC… sequence, may result in the formation of a stacking fault. Dislocation motion primarily occurs through slip and climb, and a higher SFE increases the energy barrier for stacking fault formation, thereby impeding dislocation mobility. In the context of grain refinement in the carburized layer, restricted dislocation slip hinders critical processes, such as dislocation intersection and rearrangement, ultimately obstructing subgrain formation [21,22].

Currently, no studies have been reported on the stacking fault energy (SFE) calculation of austenite in M50NiL steel. In recent years, the influence of alloying elements and carbon (C) on austenite SFE has been widely debated [23,24,25,26]. Therefore, it is essential to evaluate the SFE of austenite in M50NiL steel, investigate the effects of C atoms and alloying elements on SFE, and examine the relationship between SFE and the formation of subgrains and twins in sub-micron crystalline regions.

First-principles calculations based on quantum mechanics offer a powerful tool for analyzing the electronic structure and properties of materials. By constructing supercell models containing stacking faults and computing the energy difference compared with perfect crystals, the stacking fault energy can be determined [27,28,29].

In this study, a first-principles approach was employed to establish an austenite stacking fault model for M50NiL steel. The effects of the number and position of C atoms and alloying elements on the generalized stacking fault energy (GSFE) were systematically investigated. Additionally, differential charge density analysis was performed to clarify the underlying mechanisms by which these elements could influence SFE. The simulation results demonstrated a correlation between GSFE and the formation of subgrains and twins in the sub-micron crystalline regions of the carburized layer.

## 2. Methods

### 2.1. First-Principles Calculations

First-principles calculations were conducted using the CASTEP module [30], based on density functional theory (DFT) and employing the Perdew-Burke-Ernzerhof (PBE) exchange-correlation functional within the generalized gradient approximation (GGA) framework [31,32]. OTFG ultrasoft pseudopotentials corresponding to the PBE functional were utilized [33]. Following the convergence tests, a plane-wave cutoff energy of 550 eV and maximum of 100 electronic iterations were adopted. The total energy convergence threshold was set at 10^−6^ eV/atom. Due to the varying number of atoms extended along different directions in the supercell, a k-point mesh of 8 × 8 × 8 was adopted for single-cell calculations, whereas a 10 × 3 × 1 grid was applied for supercell calculations.

Both nonmagnetic (NM) and antiferromagnetic (AFM) configurations were considered during the simulation. The results indicated minimal differences in the SFE between the magnetic states. Similar observations were reported by N.I. Medvedeva et al. [34], who discovered only slight variation in SFE between the ferromagnetic and nonmagnetic states. Given the computational complexity of simulating magnetic multilayer supercells, the GSFE calculations in this study were conducted under a nonmagnetic configuration, which is consistent with previous studies [35]. For the relaxed FCC-Fe single-cell model, the lattice parameters were determined to be a = b = c = 3.48 Å.

The generalized stacking fault energy (γ) of austenite was quantified using the slab model approach. This involved dividing the perfect crystal structure along a designated slip plane into two parts and displacing one part relative to the other by an arbitrary vector along the interface. The energy increase per unit area resulting from this displacement was defined as γ and calculated using the following Equation [36]:

(1) $$\gamma =\frac{E-E_{0}}{A}$$

In the formula, E represents the energy of the crystal structure at any arbitrary displacement, E_0_ is the energy of the intact crystal structure, and A is the area of the stacking fault.

**Figure 1:**
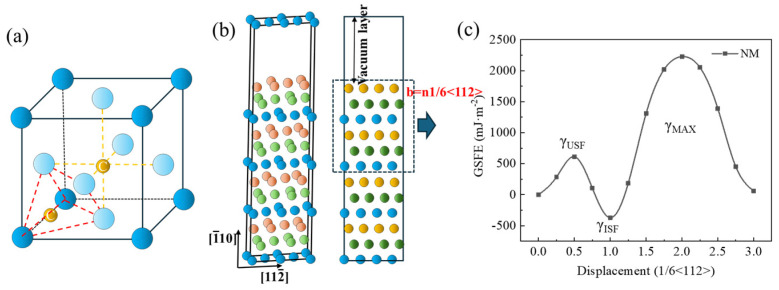
**Crystal Model of Face-Centered Cubic Fe Atoms and Calculation of Stacking Fault Energy:** (**a**) Interstitial dissolution of C atoms in the face-centered cubic Fe unit cell. (**b**) Supercell model. (**c**) Generalized stacking fault energy of face-centered cubic pure Fe.

Based on the aforementioned method for calculating γ, simulations were conducted using the (111) close-packed plane of an FCC-Fe crystal as the slip plane, with shearing performed along the [110] and [112] crystallographic directions (Figure 1b). A 2 × 1 × 4 supercell model comprising 12 atomic layers arranged in the ABCABCABC… stacking sequence was constructed, containing a total of 48 Fe atoms. The top six layers were displaced relative to the bottom six layers along the [112] direction. In face-centered cubic (FCC) structures, when the Burgers vector of a partial dislocation is *b* = 1/6 [112], an intrinsic stacking fault can be formed, corresponding to the stable stacking fault energy (γ_ISF_). A lower γ_ISF_ value indicates a reduced energy requirement for stacking fault formation and is associated with suppression of the γ→α phase transformation [34]. When *b* = 1/2 [112], the system can reach an unstable stacking fault energy (γ_USF_), which corresponds to the energy maximum along the slip path. A smaller γ_USF_ signifies a lower barrier to dislocation nucleation and thus facilitates dislocation slip [37]. The energy difference Δγ = γ_USF_ − γ_ISF_ represents the energy barrier for stacking fault formation, that is, the energy that should be overcome for the dislocations to form stacking faults [38]. Additionally, for *b* = 31 [112], the calculated energy can be referred to as the unstable twin fault energy (γ_MAX_), reflecting the energy required for twin formation in the crystal lattice [39]. The stacking fault energy values computed for the pure FCC Fe model are shown in Figure 1c. The results showed that γ_USF_ = 612.53 mJ/m^2^, γ_ISF_ = −371.27 mJ/m^2^, and γ_MAX_ = 2228.17 mJ/m^2^. These values are in close agreement with those reported by N.I. Medvedeva et al. [34], thereby validating the accuracy of the simulation model.

In the FCC Fe unit cell, interstitial C atoms can occupy either octahedral or tetrahedral sites (Figure 1a). Structural optimizations were performed for both configurations, and the total energies of the optimized structures were calculated. The octahedral interstitial configuration yielded a total energy of −3607.8131 eV, whereas the tetrahedral configuration had a higher energy of −3603.8887 eV. These results indicate that octahedral sites provide a more thermodynamically stable environment for interstitial C atoms. Therefore, in all supercell models utilized in this study, C atoms were placed in octahedral interstitial positions. The atomic diameter of C is 1.54 Å, whereas the octahedral interstitial site diameter in the matrix is approximately 1.02 Å. Consequently, the dissolution of C atoms induces lattice dilation due to the significant size misfit. In contrast, the atomic diameters of the alloying elements are Mo = 2.725 Å, V = 2.622 Å, Ni = 2.492 Å, and Cr = 2.498 Å, all of which dissolve substitutionally.

### 2.2. Heat Treatment Process

The material used in this experiment was high-purity M50NiL bearing steel produced through vacuum induction melting (VIM) followed by vacuum arc remelting (VAR). The chemical composition of steel is listed in Table 1.

The carburizing process was conducted using a low-pressure vacuum horizontal furnace (ECM) at a temperature of 960 °C with a 1:1 mixture of acetylene and nitrogen as the carburizing atmosphere. The acetylene flow rate was maintained at 33.33 sccm. A multi-stage pulsed carburizing and diffusion strategy was employed, wherein each strong carburizing stage lasted for 2 min, and the diffusion time increased progressively. The total carburizing duration was 35 h, during which the large-sized M_7_C_3_ phases were formed at recrystallized sites. Subsequently, high-pressure nitrogen gas (1.5 bar) was introduced for cooling. The post-carburizing quenching process involved holding at 850 °C for 30 min, followed by heating to the quenching temperature and holding for 40 min to ensure a uniform thermal distribution across the workpiece. The quenching temperature was optimized in the range of 1025–1150 °C, after which nitrogen gas cooling was performed at a pressure of 2 bar. Following quenching, the SMCR were formed in the near-surface layer of the carburized layer. The cryogenic treatment involved holding at −75 °C for 2 h, followed by three tempering cycles at 545 °C for 2 h each.

TEM samples were taken in the SMCR of the carburized layer by focused ion beam (FIB), and the microstructure was observed at 200 kV using a transmission electron microscope (TEM, FEI Talos F200X, Thermo Fisher Scientific, Waltham, MA, USA).

## 3. Results

### 3.1. Microstructure of M50NiL Steel After Carburizing and Quenching

**Figure 2:**
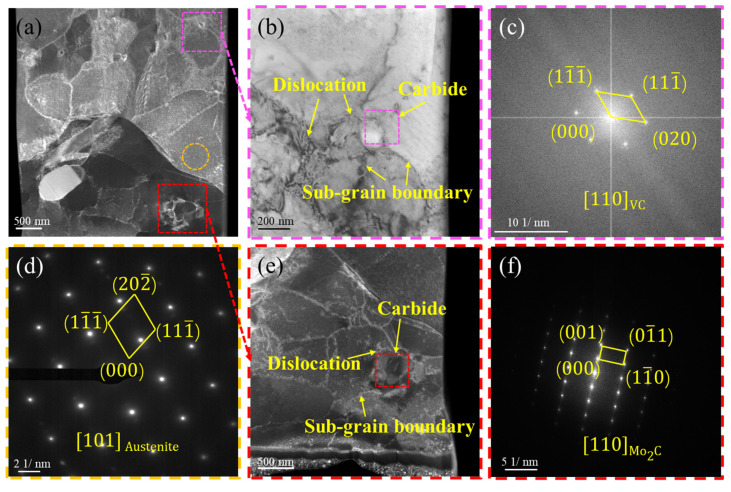
**TEM Analysis of the SMCR**: (**a**) TEM morphology analysis of the SMCR. (**b**) Bright-field image of carbides and their vicinity. (**c**) Fast Fourier transform pattern of carbide in Figure (**b**). (**d**) Diffraction pattern analysis of the matrix in Figure (**a**). (**e**) Bright-field image of carbides and their vicinity. (**f**) Fast Fourier transform of carbide in Figure (**e**).

Using the aforementioned grain refinement approach, sub-micron grains were successfully produced within the carburized layer. The FIB-TEM analysis of the SMCR is presented in Figure 2a, where numerous subgrains were observed within the original austenite grains. Figure 2b,e illustrate the carbides located at different sites, demonstrating that subgrains can form around these carbides. This observation indicated that the subgrain formation resulted from the dislocations pinned and entangled by nanoscale carbides, with a high density of dislocations near these carbide particles. The Fourier transform diffraction patterns of the carbides confirmed that the nanoscale carbides were primarily VC and Mo_2_C phases. The diffraction pattern of the matrix (Figure 2d) revealed that the matrix was austenitic and contained no observable stacking faults. This demonstrated that extended carburizing increased the carbon content in the matrix, significantly lowering the Ms temperature. Consequently, the matrix remained fully retained austenite after quenching. The stability of this retained austenite was attributed not only to the depressed Ms point but also to the presence of alloying elements, which can be further interpreted through the γ_ISF_ of the austenite. Furthermore, the absence of stacking faults in austenite indicated that the stacking fault energy was sufficiently high to inhibit their formation. Thus, the influence of stacking faults on dislocation motion can be excluded, allowing an independent assessment of the effect of stacking fault energy on dislocation dynamics.

### 3.2. Calculation of GSFE of Austenite in M50NiL Steel

**Figure 3:**
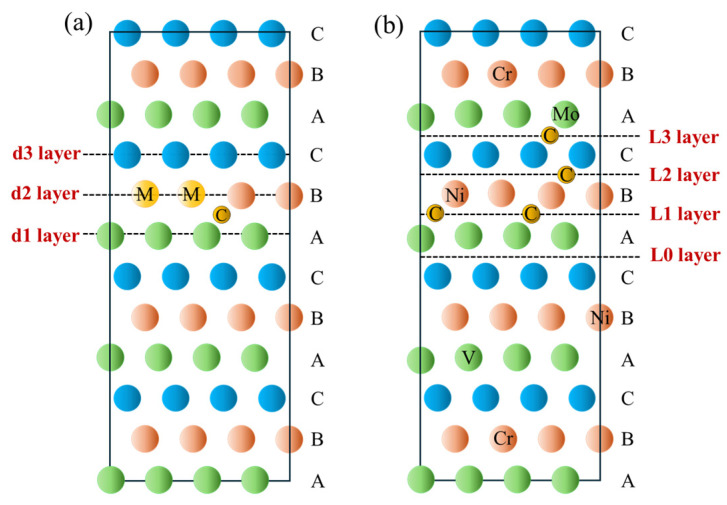
**Stacking Fault Energy Models of M50NiL Steel**: (**a**) Stacking fault energy models for different alloying elements. (**b**) Stacking fault energy models of austenite in M50NiL steel with different positions and quantities of C atoms.

Based on the model depicted in Figure 1b, the GSFE of austenite in M50NiL steel was calculated. The analysis focused on examining the effects of different alloying element types as well as the position and quantity of C atoms on the GSFE. To evaluate the influence of alloying elements, a single Fe atom in the model was replaced by an atom of a specific alloying element. Due to the substantial increase in computational cost associated with larger atom counts, the model was minimized to the smallest feasible size. Two scenarios were considered for the dissolution of C atoms in the interstitial sites of the M-Fe lattice, as shown in Figure 3a. When C atoms were interstitially located in the L0 layer, the structure exhibited significant instability, significantly deviating from the configuration of the original supercell model. This instability caused a sharp increase in the total system energy and a substantial increase in the energy barrier (with γ_USF_ − γ_ISF_ = 13,040.5152 mJ/m^2^). These results indicated that the excessively high energy barrier under this condition inhibited the nucleation of partial dislocations, thereby preventing the formation of stacking faults. Therefore, the scenario in which C atoms were located in the L0 layer was excluded from further analysis.

#### 3.2.1. Influence of Alloying Elements

**Figure 4:**
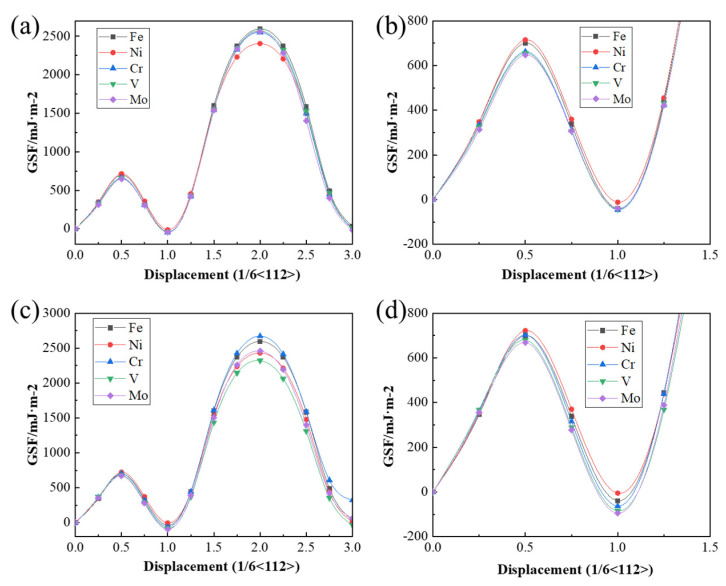
**GSFE for Different Alloying Elements**: (**a**) GSFE calculation when C atoms are not dissolved in the M-Fe interstitial sites. (**b**) Magnified view of a local region in Figure 4a. (**c**) GSFE calculation when C atoms are dissolved in the M-Fe interstitial sites. (**d**) Magnified view of a local region in Figure 4c.

The effects of different alloying elements on the generalized stacking fault energy (GSFE) were calculated (Figure 4). When C atoms were not interstitially dissolved in the M-Fe lattice, the GSFE results are presented in Figure 4a,b. Following the substitution of Fe atoms with alloying element atoms, both γ_USF_ and γ_ISF_ decreased in all systems, except for the Ni-substituted system, with the Mo-substituted system exhibiting the lowest values. Specifically, the γ_USF_ and γ_ISF_ were 700.34 and −39.56 mJ/m^2^ for the Fe system, 715.00 and −12.10 mJ/m^2^ for the Ni system, and 647.90 and −39.80 mJ/m^2^ for the Mo system, respectively.

When C atoms were interstitially incorporated into the M-Fe lattice, the GSFE values are shown in Figure 4c,d. The influence of alloying elements on USF and ISF exhibited similar trends. For instance, in the Ni system, γ_USF_ increased to 721.91 mJ/m^2^ and γ_ISF_ to −5.59 mJ/m^2^, whereas in the Mo system, γ_USF_ and γ_ISF_ were 669.08 and −95.49 mJ/m^2^, respectively. However, no consistent pattern was observed regarding the influence of alloying element type and position on γ_MAX_. For ease of comparison, the values of γ_USF_, γ_ISF_, and γ_MAX_ for each model are summarized in Table 2. These results indicated that the addition of alloying elements generally reduced the GSFE of the system. When C atoms were interstitially dissolved in the M-Fe lattice, Δγ generally increased, implying a higher energy barrier for dislocation slip. However, given the limited number of M atoms, the likelihood of C atoms occupying M-Fe interstitial sites was relatively low. Therefore, the data from configurations without C incorporation better reflected realistic conditions. Under these conditions, alloying elements, including Ni, were shown to reduce the system’s Δγ. In summary, the presence of alloying elements effectively lowered the stacking fault energy and energy barrier in austenite, thereby facilitating dislocation slip in M50NiL steel.

#### 3.2.2. Influence of the Position and Quantity of C Atoms

The grain refinement process employed in this study involved prolonged carburizing, which significantly increased the carbon content of the austenitic matrix. To investigate the effects of the position and quantity of C atoms, the positions of the alloying elements were fixed, and their atomic proportions were determined based on the atomic ratios obtained from SEM-EDS analysis of the austenitic matrix. In a 49-atom model, the approximate composition was Cr2Mo1Ni2V1Fe43. The spatial arrangement of alloying elements was based on the energy associated with C atom dissolution into M-Fe interstitial sites (with the order Mo > Cr > Fe ≈ V ≈ Ni), where the lower energy positions were placed farther from the L0 layer (i.e., the stacking fault plane) (Figure 3b). The specific positions of alloying atoms were determined based on the Special Quasi-random Structure (SQS) method, with the Warren-Cowley short-range order (SRO) parameters maintained below 0.01. To isolate the influence of carbon, the C atoms in the model were dissolved interstitially only within the Fe lattice. The stacking fault was designated to occur in the L0 layer, with subsequent layers denoted as L1, L2, etc. C atoms were placed at the octahedral interstitial sites in different layers, and GSFE was calculated for various positional and quantitative configurations.

**Figure 5:**
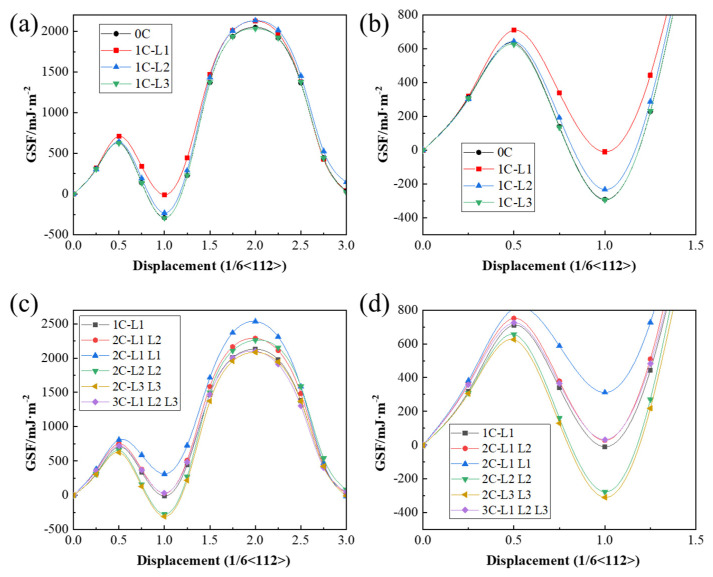
**Influence of C Atoms on the Generalized Stacking Fault Energy of the System**: (**a**) Effect of C atom positions. (**b**) Magnified view of a local region in Figure 5a. (**c**) Effect of the number of C atoms. (**d**) Magnified view of a local region in Figure 5c.

Figure 5a,b depict the GSFE results for different C atom positions. Among them, 1C-L1 indicates that there is one C atom interstitially dissolved in the L1 layer.

When C atoms were interstitially incorporated into the Fe lattice, the GSFE of the system increased significantly, particularly when C atoms were located in the L1 layer. In this case, γ_USF_ rose from 636.11 to 710.97 mJ/m^2^, and γ_ISF_ from −291.91 to −9.15 mJ/m^2^. As the position of C atom dissolution moved further from the L0 layer, GSFE gradually declined. When C atoms were placed in the L3 layer, the GSFE curve closely resembled that of the model without carbon, indicating that C atoms located far from the stacking fault layer had a negligible influence on GSFE, which was consistent with previous findings [35].

Figure 5c,d illustrate the effects of varying C atom quantities on GSFE. When C atoms were located in the L1 layer, increasing C content significantly elevated GSFE while simultaneously reducing the stacking fault formation barrier. When C atoms were in the L2 layer, the increased C content slightly increased both the stacking fault energy and barrier. In contrast, for the L3 layer, the increased C content slightly lowered the stacking fault energy and marginally increased the barrier. These findings indicated that C atoms had a minimal impact on GSFE when situated beyond the L1 layer. Therefore, the analysis was primarily focused on the C incorporation within the L1 layer. Notably, when a single C atom was located in the L1 layer, the increase in γ_ISF_ exceeded that in γ_USF_, further stabilizing the austenitic phase. This stabilizing effect became more pronounced as the number of C atoms in the L1 layer increased. The γ_USF_, γ_ISF_, Δγ and γ_MAX_ of the systems with different positions and quantities of C atoms are plotted in Table 3.

In this study, the matrix carbon content was approximately 1.3%, corresponding to approximately three C atoms in the model. When all C atoms were located far from the L0 layer, the increased C content exerted a negligible influence on GSFE. When C atoms were close to the L0 layer, the stacking fault energy increased, whereas the barrier decreased. However, given the random distribution of C atoms in the lattice in practical systems, the probability of all carbon atoms reside in the L1 layer is relatively low. As some C atoms dissolved into more distant layers, γ_USF_ and the associated barrier tended to decrease. Overall, a higher carbon content may still facilitate the dislocation slip. Compared to the pure Fe system, the γ_ISF_ of all alloyed systems was substantially elevated, which inhibited martensitic transformation. Therefore, the retained austenite content in the carburized layer exceeded 90% after carburizing and quenching. This was attributed not only to the lowered Ms temperature resulting from the increased C content but also to the inhibitory effects of both alloying elements and carbon on the transformation of austenite to martensite [40].

## 4. Discussion

### 4.1. Differential Charge Density Calculation

**Figure 6:**
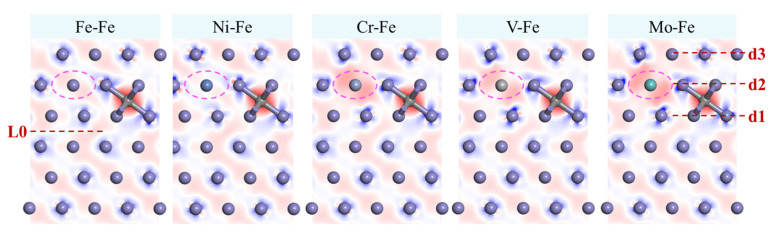
**Differential Charge Density for Different Alloying Element Systems**.

Previous studies have suggested a correlation between differential charge density and SFE [41]. Analysis of the electron distribution between atoms can facilitate the assessment of interatomic bonding and its influence on SFE [42]. Accordingly, differential charge density calculations were performed for the aforementioned models. For various alloying element systems, the analysis considered a scenario in which C atoms were interstitially dissolved in the Fe–Fe interstitial sites. The differential charge density corresponding to the formation of an intrinsic stacking fault (with a partial dislocation vector *b* = 61 ⟨112⟩) was calculated (Figure 6). In these visualizations, the red isosurfaces denote regions of charge accumulation (positive values), while the blue isosurfaces indicate regions of charge depletion (negative values). To better observe the charge distribution near the stacking fault, atoms located within the three layers above and below the L0 fault plane were analyzed. When C atoms were interstitially incorporated into the Fe–Fe interstitial sites, charge accumulation (red isosurfaces) was observed around the C atoms sourced from nearby Fe atoms. Concurrently, the charge density around neighboring Fe atoms decreased. Regarding the effects of alloying elements, all substitutional solute atoms except for Ni, such as Cr, Mo, and V, exhibited an increased charge density around their respective sites. This increase led to a corresponding decrease in the charge density of adjacent Fe atoms, notably including the second Fe atom in layer d1 and the first Fe atoms in layers d2 and d3. The Fe atoms in layer d1, closest to the stacking fault plane, experienced the most significant reduction in charge density, indicating weakening of the Fe–Fe bond in this critical region. As previously proposed by [43], the interstitial dissolution of atoms led to charge depletion around the surrounding Fe atoms, thereby weakening the Fe–Fe bonding strength and lowering the SFE. In particular, the Fe atoms in layer d1, which were closest to the stacking fault plane, significantly reduced the stacking fault energy owing to the weakening of their bonds. In the case of the Ni element, the charge density around the Ni atom was slightly reduced compared to the Fe model, which resulted in an increase in the charge density near the second Fe atom in layer d1. Consequently, γ_ISF_ of the Ni-doped system was slightly higher than that of the Fe-only system.

**Figure 7:**
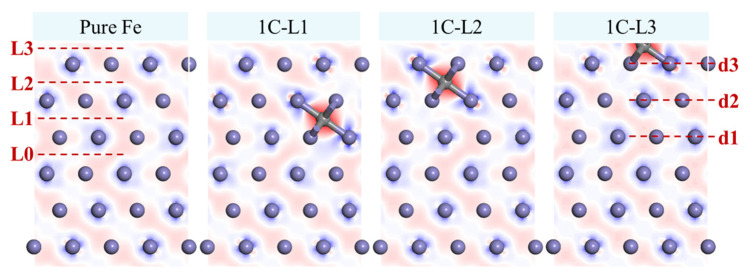
**Differential Charge Density for Systems with Different C Atom Positions**.

To isolate the effect of C atoms from that of alloying elements, differential charge density calculations were performed for a pure Fe system (Figure 7). These calculations encompassed the undoped Fe system, as well as the systems in which C atoms were interstitially incorporated into Fe interstitial sites at different layer positions. The results showed that the interstitial C incorporation markedly increased the charge density around C atoms. The corresponding changes in charge density were also evident around nearby Fe atoms. Taking the 1C-L1 model in Figure 7 as an example, the fourth Fe atom in layer d1 and the third Fe atom in layer d2 exhibited asymmetric changes in the charge distribution. On the side facing the C atom, the charge density was significantly reduced, whereas on the opposite side, it was significantly increased. This phenomenon was presumed to be associated with localized electron redistribution, possibly reflecting a compensation mechanism between charge depletion and accumulation around Fe atoms. When the C atom was located in the L1 layer, the Fe atoms in layer d1, which was the closest to the L0 stacking fault plane, exhibited a reduced charge density on the side adjacent to the C atom and an increased charge density on the side facing the L0 layer. This asymmetric distribution suggested a strengthening of Fe–Fe bonds near the L0 plane, which contributed to an increase in the stable SFE. In contrast, when the C atom was positioned in the L2 or L3 layer, changes in charge density were primarily observed in Fe atoms within layers d2 and d3, respectively. However, because of the greater distance from the L0 layer, these variations did not significantly influence the stable SFE.

### 4.2. Twinning

**Figure 8:**
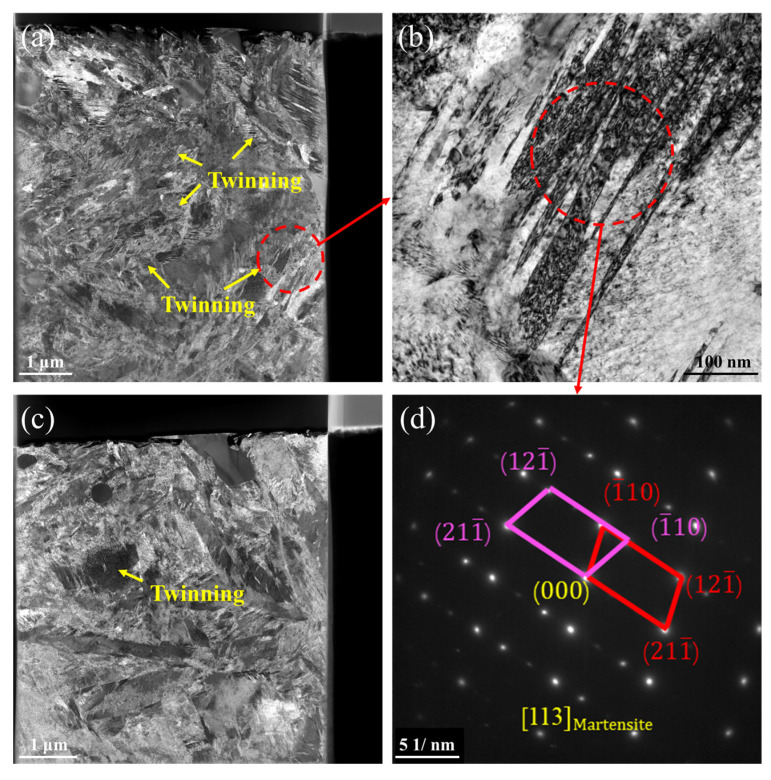
**Twinning in the Carburized Layer**: (**a**) Twin martensite in the sub-micron grain region. (**b**) Magnified view of the region in Figure 8a. (**c**) Twin martensite in the non-sub-micron grain sample. (**d**) Diffraction pattern of the twin martensite shown in Figure 8b.

After quenching, sub-micron austenite grains were generated in the carburized layer. Subsequent cryogenic and tempering treatments led to the generation of numerous twinned martensite structures (Figure 8). Both the sub-micron and non-sub-micron grain regions of the carburized layer contained twins, whereas the density of twins was notably higher in the sub-micron grain region. In the GSFE curve, γ_MAX_ denotes the unstable twin fault energy, which is closely associated with the propensity for twin formation. Based on γ_USF_, γ_ISF_, and γ_MAX_, the twinning tendency, denoted as $\tau_{a}$, can be quantitatively evaluated. The corresponding calculation formula is expressed as follows [44]:

(2) $$\tau_{a}=\left[\begin{array}{c} 1.136-0.151\frac{\gamma_{Isf}}{\gamma_{usf}} \end{array}\right]\sqrt{\frac{\gamma_{usf}}{\gamma_{MAX}}}$$

**Figure 9:**
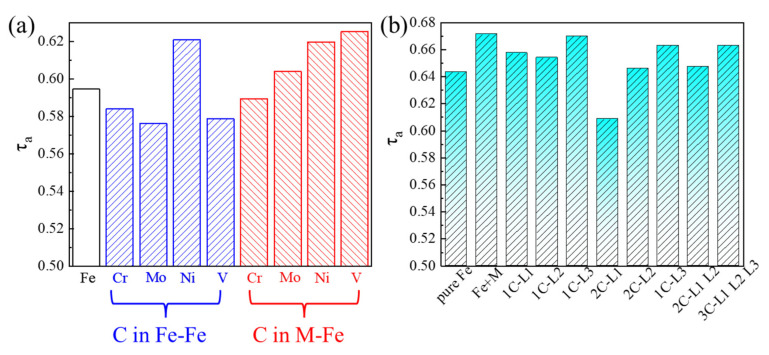
**Calculation of Twinning Tendency for Different Systems**: (**a**) Twinning tendency based on the model in Figure 3a. (**b**) Twinning tendency for the M50NiL steel stacking fault energy model in Figure 3b.

Based on Equation (2), $\tau_{a}$ of the aforementioned models was calculated (Figure 9). Figure 9a illustrates the calculations based on the model illustrated in Figure 3a. When C atoms were not interstitially dissolved in the M-Fe lattice, all alloying elements except Ni reduced twin formation tendency. In contrast, when C atoms were interstitially incorporated into the M-Fe lattice, all alloying elements increased the tendency for twin formation. For the SFE model of M50NiL steel shown in Figure 3b, the corresponding $\tau_{a}$ values are displayed in Figure 9b, with pure Fe included for comparison. The addition of all alloying elements increased the twin formation tendency from 0.64 to 0.67, indicating that these elements facilitated twin formation in M50NiL steel [45]. Regarding the influence of carbon, an overall decrease in $\tau_{a}$ was observed with increasing C content. When C atoms were located in the first layer (L1), the twin formation tendency decreased significantly. Especially when two C atoms were both situated in L1, $\tau_{a}$ decreased to 0.61. As the C atoms were positioned further from the L0 layer, the degree of reduction in $\tau_{a}$ became negligible. When C atoms were located in the L3 layer, the value of $\tau_{a}$ was nearly identical to the case without C. While increasing the number of C atoms further reduced $\tau_{a}$, the effect remained minimal.

**Figure 10:**
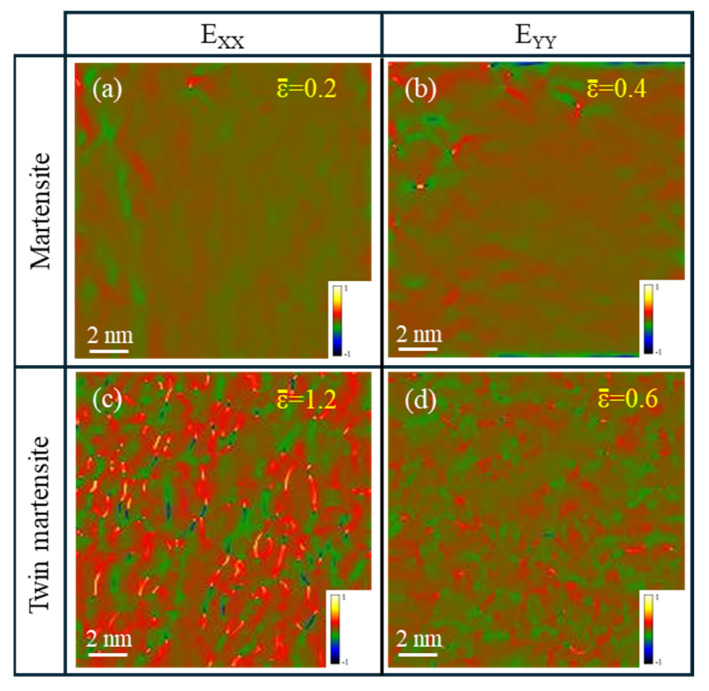
**GPA Analysis of Martensite and Twinned Martensite**: (**a**) The strain of martensite in the X direction. (**b**) The strain of martensite in the Y direction. (**c**) The strain of twinned martensite in the X direction. (**d**) The strain of twinned martensite in the Y direction.

In summary, the abundant formation of twins in the sub-micron grain region of the carburized layer could be attributed in part to the presence of alloying elements, which enhanced the twin formation tendency. Additionally, the M50NiL austenite lies in the “twinning” rather than “slip” regime at high C content. Thus, another primary factor driving twin formation was the presence of a high strain [46,47]. During quenching to room temperature, the sub-micron grain region retained austenite in a stable phase. Given the relatively low yield strength of austenite, significant plastic deformation occurred under the combined effects of thermal and transformation stresses. During cryogenic and tempering treatments, this high-strain austenite transformed into martensite, prompting atomic slip along the specific crystallographic planes and the formation of twins as a mechanism for stress relief. To further support this hypothesis, GPA was conducted on high-resolution TEM images to compare the strain levels between regular martensite and twinned martensite (Figure 10). The GPA strain distribution in the Exx and Eyy directions revealed that the twinned martensite regions exhibited significantly higher strain levels than untwinned martensite, thereby confirming that the high strain played a crucial role in twin formation. Moreover, previous studies have suggested that the rate of deformation twin formation can increase with both the strain and carbon content [48]. These findings collectively explain the substantial occurrence of twins in the sub-micron grain region of the carburized layer.

## 5. Conclusions

The SMCR was successfully produced in the carburized case of M50NiL steel via optimized carburizing heat treatment. The extensive subgrain formation in the SMCR structure stems from dislocation pinning and entanglement by nanoscale carbides. The addition of alloying elements to M50NiL steel generally reduced the unstable stacking fault energy (γ_USF_) and stacking fault formation barrier of austenite, with the exception of Ni, thereby facilitating the initiation of dislocation slip and lowering the energy required for dislocation mobility. The influence of C atoms on the generalized stacking fault energy (GSFE) of austenite was strongly dependent on their position and concentration: when C atoms were located in the L1 layer, the GSFE increased while the stacking fault formation barrier was significantly reduced, and this effect diminished as C atoms moved farther from the L1 layer. Differential charge density analyses revealed that the γ_USF_ value in austenite was closely related to the strength of Fe–Fe bonds near the L0 layer; specifically, all alloying elements except Ni weakened the Fe–Fe bonding near the stacking fault plane through substitutional solid solution behavior, resulting in decreased γ_USF_, whereas interstitial C atoms caused a redistribution of charge density due to their high electronegativity—charge depletion occurred in Fe atoms away from the L0 layer and charge accumulation in those near the L0 layer—thereby strengthening Fe–Fe bonds and increasing γ_USF_. The formation of numerous twins in the sub-micron grain region of the carburized layer arose from two primary factors: first, the addition of alloying elements enhanced twin formation tendency, and second, the stable retained austenite in this region experienced significant thermal- and transformation-induced strain during quenching, which promoted the development of martensitic twins during subsequent cryogenic and tempering treatments. Ultimately, the combined effects of alloying elements and carbon substantially increased the stable stacking fault energy, stabilizing the retained austenite phase during quenching, while simultaneously maintaining γ_USF_ and the stacking fault formation barrier at low levels to facilitate dislocation slip and reduce the energy required for dislocation motion, thereby creating favorable conditions for subgrain formation.

## Figures and Tables

**Table 1:** Chemical composition of M50NiL steel [8].

Element	C	Cr	Mo	Ni	V	Mn	Si	Fe
wt%	0.13	4.1	4.2	3.4	1.2	0.13	0.18	Bal.

**Table 2:** γ_USF_, γ_ISF_, Δγ, and γ_MAX_ for different alloying elements.

GSFE	Non-M-Fe				M-Fe			
	γ_USF_	γ_ISF_	Δγ	γ_MAX_	γ_USF_	γ_ISF_	Δγ	γ_MAX_
Fe	700.34	−39.56	739.90	2596.03	700.34	−39.56	739.90	2596.03
Cr	662.02	−45.61	707.63	2550.34	702.20	−63.30	765.50	2671.43
Mo	647.90	−39.80	687.70	2561.28	669.08	−95.49	764.57	2458.26
Ni	715.00	−12.10	727.10	2405.66	721.91	−5.59	727.50	2431.42
V	655.74	−43.05	698.79	2572.45	680.41	−85.25	765.66	2320.95

**Table 3:** γ_USF_, γ_ISF_, Δγ and γ_MAX_ under different positions and quantities of C atoms.

GSFE	C Atoms Were Not Interstitially Dissolved in the M-Fe Lattice			
	γ_USF_	γ_ISF_	Δγ	γ_MAX_
Pure Fe-0C	612.53	−371.27	983.80	2228.17
Fe-0C	636.11	−291.91	928.02	2048.90
1C-L1	710.97	−9.15	720.12	2128.88
1C-L2	644.02	−233.11	877.13	2132.99
1C-L3	626.34	−294.47	920.81	2032.84
2C-L1 L1	808.25	312.00	496.25	2533.74
2C-L2 L2	655.97	−276.63	932.6	2260.60
2C-L3 L3	625.33	−308.90	934.23	2083.88
2C-L1 L2	750.92	28.55	722.39	2287.83
3C-L1 L2 l3	724.31	32.97	691.34	2099.63

## Data Availability

The original contributions presented in this study are included in the article. Further inquiries can be directed to the corresponding author.

## References

[1] Zeng G., Liang Y., Li S., Sun Y., Wang P. (2024). Effect of carbonitride on the friction and wear properties of M50NiL steel after sequential carburizing and nitriding treatment. Mater. Today Commun..

[2] Li G.-M., Liang Y.-L., Sun H., Cao Y.-G., Zhu Z.-L. (2020). Effect of pre-existing carbides prepared by different heat treatments on the nitriding behaviour during a carburizing and nitriding duplex treatment of an M50NiL steel. Surf. Coat. Technol..

[3] Guo Q., Liu H., Sun C., Liu H., Cao Y., Wang L., Cai X., Fu P., Wang P., Li D. (2023). Effectively improving the hardness-strength-toughness of carburized bearing steel via nanoprecipitates and fine grain structure. Mater. Sci. Eng. A.

[4] Lian J.-L., Zheng L.-J., Wang F.-F., Zhang H. (2018). Evolution of carbides on surface of carburized M50NiL bearing steel. J. Iron Steel Res. Int..

[5] Qian D., Wu L., Wang F., Deng S., Yin F., Jiang S. (2024). Tailored carburization gradient microstructure and enhanced wear properties of M50NiL steel via introduced prior cold rolling. Wear.

[6] Li S., Liang Y., Yu J., Liu D., Chen J., Zeng G. (2022). The high-cycle fatigue behavior of carburized M50NiL steel with high-energy modiffcation and compound inffltration: A comparative study. Surf. Coat. Technol..

[7] Su Y., Wang J., Yu X., Wang S., Xia Y., Liu L., Liu J. (2021). Effect of deep tempering on microstructure and hardness of carburized M50NiL steel. J. Mater. Res. Technol..

[8] Ding Z., Guo J., Niu J., Zhou L., Zhang X., Ma X. (2024). Fabrication and mechanical properties of micro/nano-crystalline layers in M50NiL carburized steel. Mater. Design.

[9] Tian Y., Bagchi S., Myhill L., Po G., Martinez E., Lin Y.T., Mathew N., Perez D. (2024). Data-driven modeling of dislocation mobility from atomistics using physicsinformed machine learning. npj Comput. Mater..

[10] Sills R.B., Foster M.E., Zhou X.W. (2020). Line-length-dependent dislocation mobilities in an FCC stainless steel alloy. Int. J. Plast..

[11] Proville L., Rodney D., Marinica M.-C. (2012). Quantum effect on thermally activated glide of dislocations. Nat. Mater..

[12] Liu L., Shen P., Wu X., Wang R., Li W., Liu Q. (2017). First-principles calculations on the stacking fault energy, surface energy and dislocation properties of NbCr2 and HfCr2. Comput. Mater. Sci..

[13] Wei X.-M., Zhang J.-M., Xu K.-W. (2007). Generalized stacking fault energy in FCC metals with MEAM. Appl. Surf. Sci..

[14] Garg P., Jones M.R., Peterson N.E., Clarke A.J., Beyerlein I.J. (2025). Role of temperature on screw dislocation dynamics in Ta, W, and Ta–W alloy. J. Mater. Res. Technol..

[15] Zhang Y., Wang J., Shan H., Zhao K. (2015). Strengthening high-stacking-fault-energy metals via parallelogram nanotwins. Scr. Mater..

[16] Celebi O.K., Gengor G., You D., Mohammed A.S.K., Bucsek A., Sehitoglu H. (2024). Distorted dislocation cores and asymmetric glide resistances in titanium. Acta Mater..

[17] Huang J., Xing H., Sun J. (2012). Structural stability and generalized stacking fault energies in b Ti–Nb alloys: Relation to dislocation properties. Scr. Mater..

[18] Yu C., Okita T., Kawabata T., Uranaka S., Li X. (2025). The effects of alloying elements on hydrogen-affected generalized stacking fault energy and dislocation behavior in nickel-based alloys. Mater. Chem. Phys..

[19] Liu T., Mukhopadhyay S., Li C.-H., Li T., Ren Y., Singh P., Devaraj A. (2025). The impact of nickel concentration and stacking fault energy on deformation mechanisms in high-purity austenitic Fe-Cr-Ni alloys. Mater. Charact..

[20] Zhang S. (2025). The Peierls stress of interfacial dislocation under lattice mismatches. J. Appl. Phys..

[21] Xue C., Gao B., Han T., Che C., Chu Z., Tuo L. (2024). Dislocation evolution mechanism of plastic deformation process of AZ31 magnesium alloy with different grain size. Comput. Mater. Sci..

[22] Qian D., Chen J., Luo H., Wang F., Hua L. (2024). Electric current-induced directional slip of dislocation and grain boundary ordering. Mater. Today Adv..

[23] Song J., Fu Y. (2023). First-principles calculation of stacking fault energies in Ni2(Cr, Mo). Mater. Today Commun..

[24] Breidi A., Allen J., Mottura A. (2024). First-principles calculations of intrinsic stacking fault energies and elastic properties in binary nickel alloys. Materialia.

[25] Brodie J., Ghazisaeidi M. (2023). First-principles calculations of the temperature dependence of stacking fault energies in Mg. Scr. Mater..

[26] Zhao Y.X., Huang Y.C., Liu Y. (2022). Insight into the stacking fault energy, dislocation, and thermodynamic properties of L12-Al3X(X=Sc, Ti, V) intermetallics from ffrst-principles calculations. Mater. Today Commun..

[27] Huang H., Shao L., Liu H. (2021). Stacking fault energies of high-entropy nitrides from ffrst-principles calculations. Solid State Commun..

[28] Wang C., Wu H., Zhu H., Sun Y.-T., Xie C. (2021). The effects of impurities on the generalized stacking fault energy of InP by first-principles calculation. Mater. Lett..

[29] Yu H., Cao S., Youssef S.S., Ma Y.-J., Lei J.-F., Qi Y., Hu Q.-M., Yang R. (2021). Generalized stacking fault energies and critical resolved shear stresses of random a-Ti-Al alloys from first-principles calculations. J. Alloys Comp..

[30] Clark S.J., Segall M.D., Pickard C.J., Hasnip P.J., Probert M.I.J., Refson K., Payne M.C. (2005). First principles methods using CASTEP. Z. Krist..

[31] Kresse G., Furthmuller J. (1993). Ab initioo molecular dynamics for open-shell transition metals. Phys. Rev. B.

[32] Hohenberg P., Kohn W. (1964). Inhomogeneous electron gas. Phys. Rev. Lett..

[33] Perdew J.P., Burke K., Ernzerhof M. (1996). Generalized gradient approximation made simple. Phys. Rev. Lett..

[34] Medvedeva N., Park M., Van Aken D., Medvedeva J. (2014). First-principles study of Mn, Al and C distribution and their effect on stacking fault energies in fcc Fe. J. Alloys Compd..

[35] Abbasi A., Dick A., Hickel T., Neugebauer J. (2011). First-principles investigation of the effect of carbon on the stacking fault energy of Fe–C alloys. Acta Mater..

[36] Vitek V. (1968). Intrinsic stacking faults in body-centred cubic crystals. Phil. Mag..

[37] Chen Z., Zhu H., Cao Y., Liu H., Zhao Z., Chen X., Li D. (2025). Revisiting the effect of localized alloying elements on stacking fault energy in austenitic steel. Mater. Sci. Eng. A.

[38] Wu X.-Z., Wang R., Wang S.-F., Wei Q.-Y. (2010). Ab initio calculations of generalized-stacking-fault energy surfaces and surface energies for FCC metals. Appl. Surf. Sci..

[39] Kibey S., Liu J.B., Johnson D.D., Sehitoglu H. (2006). Generalized planar fault energies and twinning in Cu–Al alloys. Appl. Phys. Lett..

[40] Dumay A., Chateau J.-P., Allain S., Migot S., Bouaziz O. (2008). Influence of addition elements on the stacking fault energy and mechanical properties of an austenitic Fe–Mn–C steel. Mater. Sci. Eng. A.

[41] Zhang X.Y., Min X.H., Lu C. (2024). Effects of N, O, S on generalized stacking fault energies and dislocation movements in γ-Ni and γ′-Ni3Al. Comput. Theor. Chem..

[42] Li Y., He Y., Liu S., Wang Y., Ma X. (2024). First-principles study the effect of hydrogen atoms on the generalized stacking fault energy in γ-Fe. Int. J. Hydrogen Energy.

[43] Zhu L.Y., Wu Z.X. (2023). Effects of short range ordering on the generalized stacking fault energy and deformation mechanisms in FCC multiprincipal element alloys. Acta Mater..

[44] Kivy M.B., Zaeem M.A. (2017). Generalized stacking fault energies, ductilities, and twinnabilities of CoCrFeNi-based face-centered cubic high entropy alloys. Scr. Mater..

[45] Sohrabi M.J., Naghizadeh M., Mirzadeh H. (2020). Deformation-induced martensite in austenitic stainless steels: A review. Arch. Civ. Mech. Eng..

[46] Man J., Obrtlík K., Petrenec M., Beran P., Smaga M., Weidner A., Dluhoš J., Kruml T., Biermann H., Eifler D. (2011). Stability of austenitic 316L steel against martensite formation during cyclic straining. Procedia Eng..

[47] Hasan S.M., Ghosh A., Chakrabarti D., Singh S.B. (2023). Deformation-induced martensite transformation and variant selection in AISI 316L austenitic stainless steel during uniaxial tensile deformation. Mater. Sci. Eng. A.

[48] Li S., Hu G., Jing B., Zhao Q., Su S., He M., Wei Z., Tian Y., Wang C., Ping D. (2022). Dependence of {112}<111>-type twin density on carbon content in Fe-C martensite. J. Mater. Res. Technol..

